
JOURNAL INFORMATION
==============================
NLM Title Abbreviation: Orthop Surg
Journal ID: Orthop Surg
NLM Journal ID: 101501666
Journal ID: OS

Orthopaedic Surgery

ISSN: 1757-7853
EISSN: 1757-7861
Publisher: Wiley

ARTICLE INFORMATION
==============================
PMCID: PMC11474497
DOI: 10.1111/os.13346
Article ID: OS13346
Article version: 1
Subjects: Issue Information, Issue Information

Issue Information

Electronic publication date: 2023 Dec 3
Publication date: 2023 Dec
Volume: 15
Issue: 12
First page: 3013
Last page: 3014
Copyright: © 2023 The Authors. Orthopaedic Surgery published by Tianjin Hospital and John Wiley & Sons Australia, Ltd.
License: This is an open access article under the terms of the http://creativecommons.org/licenses/by/4.0/ License, which permits use, distribution and reproduction in any medium, provided the original work is properly cited.
License URL: https://creativecommons.org/licenses/by/4.0/

Figure count: 0
Table count: 0
Page count: 9

source-schema-version-number: 2.0
cover-date: December 2023
details-of-publishers-convertor: Converter:WILEY_ML3GV2_TO_JATSPMC version:6.4.9 mode:remove_FC converted:15.10.2024

==============================

Volume 15 • Issue 12 • December 2023 • ISSN 1757-7861

### Orthopaedic Surgery

Open Access

REVIEW ARTICLES

3015
EMERGING ROLES OF RNA BINDING PROTEINS IN INTERVERTEBRAL DISC DEGENERATION AND OSTEOARTHRITIS

3026
COLLAGEN-BASED HYDROGELS FOR CARTILAGE REGENERATION

3046
ADVANCES IN THE APPLICATION OF BONE TRANSPORT TECHNIQUES IN THE TREATMENT OF BONE NONUNION AND BONE DEFECTS

CLINICAL ARTICLES

3055
RISK FACTORS FOR DISTAL JUNCTIONAL PROBLEMS FOLLOWING LONG INSTRUMENTED FUSION FOR DEGENERATIVE LUMBAR SCOLIOSIS: ARE THEY RELATED TO THE PARASPINAL MUSCLES

3065
ASSESSING THE CASUAL ASSOCIATION BETWEEN SEX HORMONE LEVELS AND FRACTURE RISK: A TWO-SAMPLE MENDELIAN RANDOMIZATION STUDY

3075
PARAMENISCAL CYST ARTHROSCOPIC EXCISION BY THE MODIFIED FAT PAD APPROACH CAN SHORTEN RECOVERY TIME—A NEW SURGICAL METHOD WITH RETROSPECTIVE STUDY

3083
SEQUENTIAL CORRECTION VERSUS CONVENTIONAL CORRECTION FOR SEVERE AND RIGID KYPHOSCOLIOSIS: A RETROSPECTIVE CASE CONTROL STUDY

3092
BENEFITS OF EARLY SURGICAL TREATMENT FOR PATIENTS WITH MULTILEVEL CERVICAL CANAL STENOSIS OF ACUTE TRAUMATIC CENTRAL CORD SYNDROME

3101
TREATMENT OF DISTAL THIRD HUMERAL SHAFT FRACTURE WITH INTRAMEDULLARY NAIL COMBINED WITH ANTERIOR MINIMALLY INVASIVE PLATE OSTEOSYNTHESIS

3108
CEMENT-AUGMENTED SCREW FIXATION WITH PHILOS PLATING FOR OSTEOPOROTIC PROXIMAL HUMERAL FRACTURES: AN OBSERVATION OF MID- AND LONG-TERM CURATIVE EFFICACY

3118
APPLY ANTICOAGULANTS AFTER DISCHARGE NOT BENEFIT TO THE INCIDENCE OF SYMPTOMATIC THROMBUS IN HIGH-ALTITUDE AREAS: A RETROSPECTIVE STUDY

3126
CORTICAL ENDPLATE BONE DENSITY MEASURED BY NOVEL PHANTOMLESS QUANTITATIVE COMPUTED TOMOGRAPHY MAY PREDICT CAGE SUBSIDENCE MORE CONVENIENTLY AND ACCURATELY

3136
DIABETES-RELATED SCREW LOOSENING: THE DISTINCTION OF SURGICAL SITES AND THE RELATIONSHIP AMONG DIABETES, IMPLANT STABILIZATION AND CLINICAL OUTCOMES

3146
INTRA-OPERATIVE NEUROPHYSIOLOGICAL MONITORING IN PATIENTS UNDERGOING POSTERIOR SPINAL CORRECTION SURGERY WITH PRE-OPERATIVE NEUROLOGICAL DEFICIT: ITS FEASIBILITY AND HIGH-RISK FACTORS FOR FAILED MONITORING

3153
CONSECUTIVE CASE SERIES OF UNIPORTAL FULL-ENDOSCOPIC UNILATERAL LAMINOTOMY FOR BILATERAL DECOMPRESSION IN LUMBAR SPINAL STENOSIS: RELATIONSHIP BETWEEN DECOMPRESSION RANGE AND FUNCTIONAL OUTCOMES

3162
A NOVEL INTRAOPERATIVE POSTURE-ADJUSTMENT APPARATUS FOR CORRECTION OF CERVICAL LORDOSIS IN ANTERIOR CERVICAL SURGERY

3174
HOW DO THE MORPHOLOGICAL ABNORMALITIES OF FEMORAL HEAD AND NECK, FEMORAL SHAFT AND FEMORAL CONDYLE AFFECT THE OCCURRENCE AND DEVELOPMENT OF MEDIAL KNEE OSTEOARTHRITIS

3182
TREATMENT OF UNSTABLE SACRAL FRACTURES WITH ROBOTICALLY-AIDED MINIMALLY INVASIVE TRIANGULAR FIXATION

3193
REVERSE LUMBAR PEDICLE SCREW IN OBLIQUE LATERAL INTERBODY FUSION: A NOVEL CONCEPT TO RESTRICT CAGE SUBSIDENCE

3202
METAPHYSEAL METAL SLEEVES FOR RECONSTRUCTION OF SEVERE KNEE BONE DEFECTS: EXCELLENT SURVIVAL RATE AT A MEAN FOLLOW-UP OF 6.4 YEARS

3209
ANALYSIS OF POTENTIAL RISK FACTORS FOR CEMENT LEAKAGE INTO PARASPINAL VEINS AFTER VERTEBROPLASTY FOR ACUTE OSTEOPOROTIC VERTEBRAL FRACTURES BASED ON A 3D RECONSTRUCTION TECHNIQUE: A RETROSPECTIVE MATCHED CASE–CONTROL STUDY

3223
CLINICAL OUTCOMES OF MINIMALLY INVASIVE FIXATION WITH PRE-BENT ELASTIC STABLE INTRAMEDULLARY NAILS FOR THE TREATMENT OF DISTAL RADIUS METAPHYSEAL DIAPHYSIS JUNCTION FRACTURES IN CHILDREN

3231
COMPLICATION RATES AND SURVIVAL OF NONAGENARIANS AFTER HIP HEMIARTHROPLASTY VERSUS PROXIMAL FEMORAL NAIL ANTIROTATION FOR INTERTROCHANTERIC FRACTURES: A 15-YEAR RETROSPECTIVE COHORT STUDY OF 113 CASES

RESEARCH ARTICLES

3243
BIOMECHANICAL COMPARISON BETWEEN ROTATIONAL SCARF OSTEOTOMY AND TRANSLATIONAL SCARF OSTEOTOMY: A FINITE ELEMENT ANALYSIS

3254
COMPARING THE EFFECT OF SPINAL AND GENERAL ANESTHESIA FOR HIP FRACTURE SURGERY IN OLDER PATIENTS: A META-ANALYSIS OF RANDOMIZED CLINICAL TRIALS

3263
THE INFLUENCE OF SCREW POSITIONING ON CAGE SUBSIDENCE IN PATIENTS WITH OBLIQUE LUMBAR INTERBODY FUSION COMBINED WITH ANTEROLATERAL FIXATION

3272
ANALYSIS OF RISK FACTORS FOR IN-HOSPITAL DEATH IN ELDERLY PATIENTS WITH TEXAS STAGE 3 AND 4 DIABETIC FOOT ULCERS AFTER TIBIAL TRANSVERSE TRANSLATION: A CASE–CONTROL STUDY

3279
AGE-RELATED CHANGES WITH THE TRABECULAR BONE OF WARD'S TRIANGLE AND NECK-SHAFT ANGLE IN THE PROXIMAL FEMUR: A RADIOGRAPHIC STUDY

3288
PROGNOSTIC FACTORS AND SURGICAL IMPACT OF NON-METASTATIC CONVENTIONAL CHONDROSARCOMA OF THE EXTREMITIES

OPERATIVE TECHNIQUES

3300
ONE STEP DOUBLE AUGMENTATION WITH HUMAN DERMIS ALLOGRAFT AND HOMOLOGOUS PRP IN MISDIAGNOSED AND OR CHRONIC ACHILLES TENDON RUPTURES

3309
IMPLEMENTATION OF A SPECIFIC SET OF INTRAOPERATIVE C-ARM FLUOROSCOPY TERMINOLOGIES IN PERCUTANEOUS VERTEBROPLASTY

3317
A NEW TECHNOLOGY USING MIXED REALITY SURGICAL NAVIGATION WITH THE UNLOCKING CLOSED REDUCTION TECHNIQUE FRAME TO ASSIST PELVIC FRACTURE REDUCTION AND FIXATION: TECHNICAL NOTE

3326
BEST OF BOTH WORLDS? FIXATION OF DISTAL FEMUR FRACTURES WITH THE NAIL-PLATE CONSTRUCT

CASE REPORTS

3335
A NOVEL TECHNOLOGY FOR 3D-PRINTING ARTIFICIAL VERTEBRAL BODIES FOR TREATING LUMBAR SPINE ADRENAL PHEOCHROMOCYTOMA METASTASES: A CASE REPORT AND REVIEW OF THE LITERATURE

3342
TUMOR-INDUCED OSTEOMALACIA: A CASE REPORT AND ETIOLOGICAL ANALYSIS WITH LITERATURE REVIEW

WILEY

View this journal online at wileyonlinelibrary.com/journal/os

### Orthopaedic Surgery

**Editor-in-Chief**
Gui-xing Qiu, *Beijing, China*
Francis John Hornicek, *Boston, USA*

**Associate Editor**
Yong-cheng Hu, *Tianjin, China*
Thomas Blattert, *Leipzig, Germany*
Douglas Letson, *Tampa, USA*
Xin-long Ma, *Tianjin, China*
Ling Qin, *Hong Kong, China*

**Editorial Board**
Giovanni Alessi, *Gent, Belgium*
Haluk Berk, *Izmir, Turkey*
Thomas Blattert, *Leipzig, Germany*
Mathis Bostrom, *New York, USA*
Kao-wha Chang, *Taiwan, China*
Edmund YS Chao, *Baltimore, USA*
Liao-bin Chen, *Wuhan, China*
Yan-xi Chen, *Shanghai, China*
Kenneth MC Cheung, *Hong Kong, China*
Thomas L. Clemens, *Birmingham, USA*
Benny Thellefsen Dahl, *Copenhagen, Denmark*
Lian-fu Deng, *Shanghai, China*
Jean Destandau, *Bordeaux, France*
Jian Dong, *Shanghai, China*
Shun-wu Fan, *Hangzhou, China*
David Fang, *Hong Kong, China*
Shi-jun Gao, *Shijiazhuang, China*
Wei Guo, *Beijing, China*
Yong Hai, *Beijing, China*
Alan R. Hargens, *San Diego, USA*
Francis John Hornicek, *Boston, USA*
William C. Horton, *Atlanta, USA*
John Hsiang, *Seattle, USA*
Yong-cheng Hu, *Tianjin, China*
Qing Jiang, *Nanjing, China*
Paul Jutte, *Groningen, Netherlands*
Jean Pierre Kalala, *Gent, Belgium*
Choon-Ki Lee, *Seoul, Korea*
Jean Jacque Lehuec, *Bordeaux, France*
Lawrence G. Lenke, *Seattle, USA*
Douglas Letson, *Tampa, USA*
Kwok Sui Leung, *Hong Kong, China*
Jian-hao Lin, *Beijing, China*
Bao-ge Liu, *Beijing, China*
Wei-hee Lo, *Taiwan, China*
William Weijia Lu, *Hong Kong, China*
Keith DK Luk, *Hong Kong, China*
Zong-ping Luo, *Suzhou, China*
Cyril Mauffrey, *Denver, USA*
Wei-hu Ma, *Ningbo, China*
Xin-long Ma, *Tianjin, China*
Ling Qin, *Hong Kong, China*
Gui-xing Qiu, *Beijing, China*
Axel Reinhardt, *Potsdam, Germany*
Pu-yi Sheng, *Guangzhou, China*
Harry L Shufflebarger, *Miami, USA*
David Stulberg, *Chicago, USA*
Paul Sung, *San Diego, USA*
Paul Unwin, *Leicester, UK*
Luc F. De Waele, *Gent, Belgium*
Fei Wang, *Shijiazhuang, China*
Wei-jun Wang, *Nanjing, China*
James N. Weinstein, *Chicago, USA*
Hee-Kit Wong, *Singapore*
Su-jia Wu, *Nanjing, China*
Jian-ru Xiao, *Shanghai, China*
Hui-lin Yang, *Suzhou, China*
Tzai-chiu Yu, *Taiwan, China*
Xi-jie Yu, *Chengdu, China*
Hansen A. Yuan, *New York, USA*
Hong-wen Zhu, *New York, USA*

**Address for Editorial Correspondence:** Editorial Board of *Orthopaedic Surgery*, Tianjin Hospital, 122 Munan Road, Tianjin 300050, China (email: orthopaedicsurgery@126.com; telephone: +86 22 2833 4734; fax: +86 22 2824 1184).

**Disclaimer:** The Publisher, Tianjin Hospital and Editors cannot be held responsible for errors or any consequences arising from the use of information contained in this journal; the views and opinions expressed do not necessarily reflect those of the Publisher, Tianjin Hospital and Editors, neither does the publication of advertisements constitute any endorsement by the Publisher, Tianjin Hospital and Editors of the products advertised.

**Copyright © 2023 The Authors. Orthopaedic Surgery published by Tianjin Hospital and John Wiley & Sons Australia, Ltd**

**Copyright and Copying (in any format)**
Copyright © 2023 The Authors. Orthopaedic Surgery published by Tianjin Hospital and John Wiley & Sons Australia, Ltd. All rights reserved. No part of this publication may be reproduced, stored or transmitted in any form or by any means without the prior permission in writing from the copyright holder. Authorization to copy items for internal and personal use is granted by the copyright holder for libraries and other users registered with their local Reproduction Rights Organisation (RRO), e.g. Copyright Clearance Center (CCC), 222 Rosewood Drive, Danvers, MA 01923, USA (www.copyright.com), provided the appropriate fee is paid directly to the RRO. This consent does not extend to other kinds of copying such as copying for general distribution, for advertising or promotional purposes, for republication, for creating new collective works or for resale. Permissions for such reuse can be obtained using the RightsLink “Request Permissions” link on Wiley Online Library. Special requests should be addressed to: permissions@wiley.com

For submission instructions, subscription and all other information visit: http://wileyonlinelibrary.com/journal/os

ISSN 1757-7861 (Online)

OS.JEB.December23
